# The influence of study area selection and landslide inventory practices on landslides spatial distribution: an example from Northern Morocco

**DOI:** 10.1038/s41598-026-36587-y

**Published:** 2026-01-17

**Authors:** Ali Bounab, Reda Sahrane, Younes El Kharim, Oussama Obda, Mohamed Mastere, Ilias Obda

**Affiliations:** 1 Superior School of Technology, Sultan Moulay Slimane University, Fkih Ben Salah, Morocco; 2 Data4Earth Lab, FST, Sultan Moulay Slimane University, Campus Mghila, P.B. 523, Béni-Mellal, Morocco; 3 GERN, Faculté des Sciences, Abdelmalek Essaadi University, Tetouan, 93000 Morocco; 4 Scientific Institute, Mohammed V University in Rabat, Avenue Ibn Battouta, PoBOX: 703, Rabat, 10000 Morocco; 5 School of Public Management, Governance and Public Policy, College of Business & Economics, University of Johannesburg, Auckland Park Kingsway Campus, Johannesburg, 2092 South Africa; 6 École Supérieure De L’Education et de la Formation Oujda, Oujda, Morocco

**Keywords:** Landslides susceptibility, Size-frequency distribution, Geomorphology, Study area, Northern Morocco, Environmental sciences, Environmental social sciences, Hydrology, Natural hazards, Planetary science, Solid Earth sciences

## Abstract

Numerous studies focused on the technical limitations of Landslides Susceptibility Maps (LSM). They were concerned with the impact of LSM technique selection, conditioning factor combinations, and/or Landslides Inventory Map (LIM) practices on LSM sensitivity. However, no previous papers focused on study area selection and its influence on the output. In fact, most authors subdivide their study area into administrative/political territories, which may be useful for decision makers but is not very informative from a pure scientific stand point. Therefore, 3 territories of Northern Morocco were investigated in this study: the first corresponds to the 1:50 000 Tetouan topographic map (cartographic), the second covers Martil watershed (geomorphological) and the third is Tetouan province (political). The latter study area is of capital importance given its two contrasted geological and morphotectonic domains (Internal and External Rif), which may produce errors in the output. The input LIM datasets for the purpose of this study are: new-active LIM, Inactive-young LIM, Relict LIM, and all landslides LIM. We used two conventional LSM algorithms (Logistic Regression and Artificial Neural Networks) in order to avoid technique-specific biases. Our results show that study area selection is not as important as LIM with regard to the output LSMs, but remains very relevant in determining LSM distribution and accuracy for Tetouan map and Martil watershed study areas. As for Tetouan province, the model is unchanged using the same LIM in the External Rif but changes significantly in the Internal Rif. Our LSM analyses also revealed the link between landslides age and elevation in the External Rif domain where relict processes are mostly concentrated in mid-slopes while new-active ones occur in lower slopes. This is not observed in the Internal Rif, which further exhibits the importance of study area selection based on naturally delimited geomorphological units rather than political or cartographic boundaries.

## Introduction

 Since the 1930’s geomorphologists had taken special interest in mass movements as a distinct geomorphological phenomenon^1–6^. Based on their work, more precise and objective classifications emerged in the late 1970’s and the early 1980’s, such as the work of^7,8^ and other geologists and geomorphologists alike. These early landslides researchers attempted to objectively classify landslides processes based on different criteria. These investigations allowed distinguishing landslides studies as a separate discipline of Earth Sciences. Since then, many researchers explored the conditioning and triggering factors that control the spatio-temporal evolution of landslides^9–15^. Such studies focus mainly on the physical aspect of landslides hazard assessment through employing several field and laboratory tests. However, due to the significant socio-economic impact of mass movements worldwide^16–19^, landslides researchers shifted their attention from physical, geological and geomorphological investigations to prediction and prevention efforts. This transition started in the early 1990’s with the adoption of conditional probability models by some researchers working in the field of earth sciences^20–22^. Although the proposed methods were initially used for mineral exploration purposes, the binary nature of the explained variables in such models made it possible to assess the statistical association between landslides and their potential conditioning factors to generate Landslides Susceptibility Maps (LSM). Subsequently, this research trend dominated the field from 2005 to 2015 as the number of research papers dealing with the subject, multiplied exponentially^23^. As a result of the global interest in this exercise, the term LSM became almost synonymous with landslides research. However, this growth came at the cost of physical and geomorphological studies especially at the regional scale where the overreliance on explanatory statistics limited the ability of researchers to properly investigate the cause-and-effect relationship between landslide processes and their causal factors.

To investigate the appropriateness of LSMs as reliable spatial prediction tools, the influence of input data and computation technique selection were investigated in previous studies. For the former category^24–26^, showed that using more predictive variables does not always produce more accurate models, while^27,28^ proved that the Landslides Inventory Map (LIM) used to train the algorithm constitutes the main control factor of LSM output. With regard to the techniques used, many researchers evaluated bivariate, multivariate and artificial intelligence methods, with different and sometimes contradictory results regarding the best technique of the bunch^29–35^.

Despite these efforts, the usefulness and reliability of LSMs as predictive tools for proper urban and strategic planning is still under-investigated, with most papers (although numerous) ignoring the predictive power of their models and focusing mainly on their goodness of fit as a quality control measure. Both accuracy assessment strategies, although similar, do not always mean the same thing as multiple factors determine the outcome of LSM accuracy assessment. In fact, the issues surrounding LSM practices include two aspects that are yet to be fully explored. The first involves the study area selection. In most cases, the analysis is conducted in areas defined by administrative and/or political boundaries (communes, provinces, regions … etc.)^26,27,36,37^. While this may be interesting from a risk management stand point, it makes little sense scientifically since such boundaries are artificial and consequently do not split the field into comprehensive and comparable geomorphological and/or morpho-tectonic units. The possible effect of such practices is the under or over estimation of statistical association between landslides and their conditioning factors. This is especially true in cases where the territory of interest houses heterogenous and sometimes contrasted slope dynamics that evolved in different geomorphological settings and morphostructural units^38^. Actually, given the probabilistic nature of LSMs, the correlation coefficient calculated for each predictive variable constitutes the basis of the landslide’s spatial predictive models. However, statisticians already consider the attenuation of correlation coefficient to be of capital importance for understanding statistical models in all kinds of scientific investigations^39^. In LSM research, this issue may occur because of the local high correlation between landslides occurrence and a specific landslides predictive variable or category that is not found elsewhere in the study area. As such, the reliability of quantitative hazard assessment models may be compromised and subjectified by this issue, which is mainly related to study area selection.

The second aspect of LSM research that deserves further investigation involves model validation practices, especially with regard to the preparation of validation datasets. A common practice in the discipline is the subset of the initial LIM into a training and a validation dataset using a random sampling technique. However, such strategies make little sense especially in areas where current and past slope processes differ significantly. In fact it is well known that climate change since Quaternary times influences slope dynamics in many areas of the world^40,41^. Given that the random sampling subset does not consider a temporal threshold for selecting validation landslides, relict processes may be used to validate LSMs while recent processes are integrated in the model computation step. Since LSM is by nature a predictive tool, such problems compromise the reliability of LSM performance assessment as a whole.

Hence, we attempt to investigate in this paper both of the above issues using a large LIM dataset that covers the entirity of Tetouan province territory. To tackle the issue of study area selection, we will calculate three LSMs, one covering the territory of Tetouan province (administrative territory), the second is delimited by the cartographic boundaries set by the 1:50 000 official Tetouan topographic map and finally the third LSM that covers the Tetouan watershed. With regards to validation, LSMs for each study area will be trained by different LIMs, some including relict processes and others excluding the latter. Then the results will be validated using landslides that occurred during the 2003–2010 rainy period. Other individual occurrences corresponding to a brief rainy period in 2018 are also integrated in the validation dataset.

## Study area

**Fig. 1:**
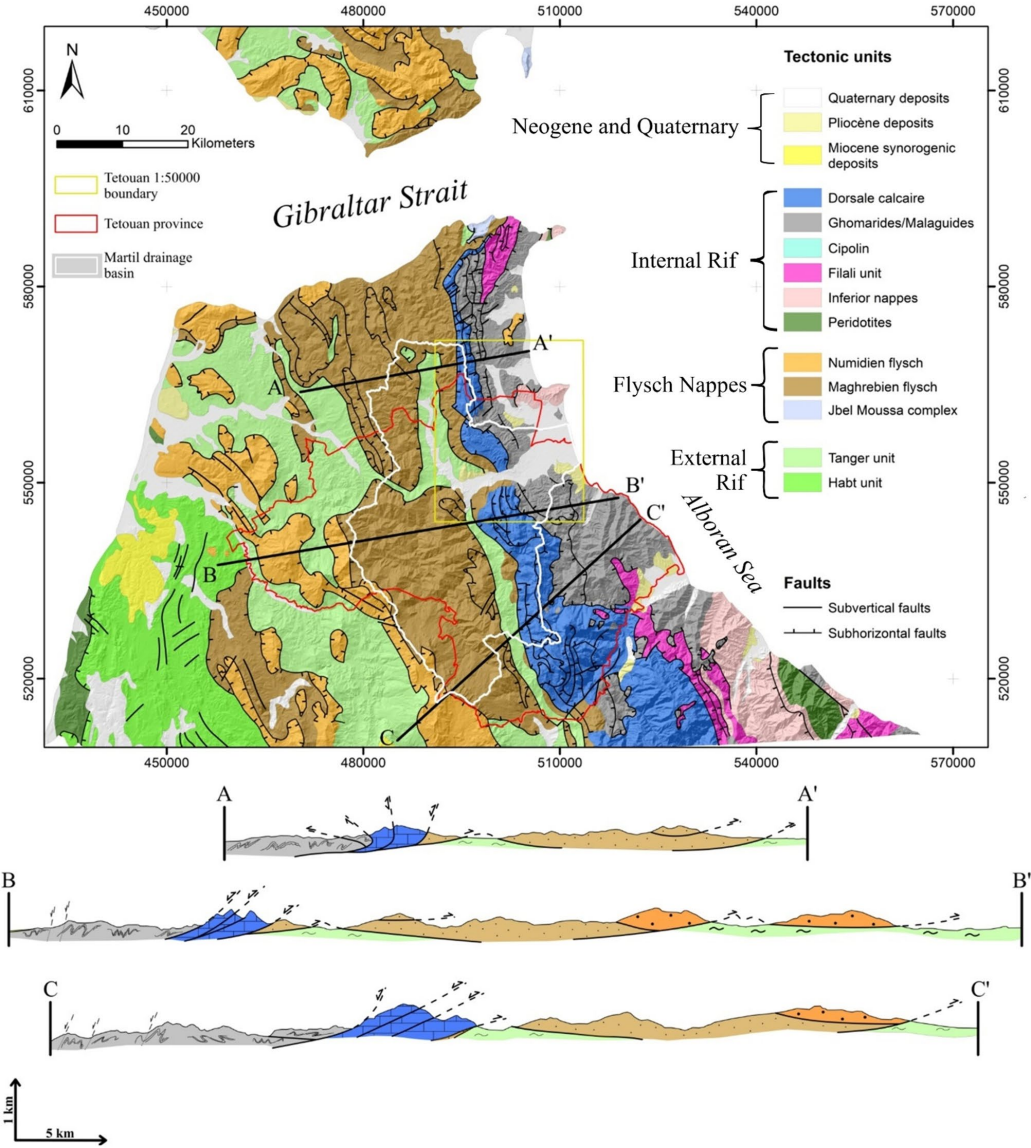
Geological map of the study area compiled and simplified from the 1:50 000 official geological map of Ksar Es-Sghir, and 2D geological cross sections first published by^53^, (used software : *ArcGIS Pro 3.5*, https://pro.arcgis.com/fr/pro-app/).

As previously stated, three overlapping territories are investigated (Fig. 1). The first is Tetouan province, which was delimited based on political/administrative boundaries. The second is the territory corresponding to the Tetouan 1:50000 official topographic map released in the late 1960’s.

With regard to the geology of these study areas of the Rif mountain range, all of the outcropping geological units can be attributed to the so-called Internal Domain or External Domain^42,43^. Both domains differ significantly with regard to their lithological facies and geological structure. For instance, while the Internal Domain is mainly dominated by Paleozoic formations that underwent various degrees of metamorphism^44–46^, the External Domain is mainly formed by late Cretaceous to early Miocene sedimentary rocks that exclusively show evidence of anchi-epizonal metamorphism in the Ketama formations^47–50^. Nevertheless, the latter tectonic unit does not outcrop in our study area, thus the External Domain is considered to be solely formed of sedimentary rocks for the purpose of this study. In addition, the thrust sheet structure of the Rif chain is more evident in the external domain where the lithological diversity of the sedimentary rocks outcropping alongside thrust faults, induces intense differential erosion processes. The latter have less effect on the mostly homogenous Paleozoic formations. On the other hand, normal faulting and vertical displacements related to neotectonic deformation are more prevalent in the Internal Domain. This is especially true near the coastline as a result of the Alboran Sea genesis and the Sebtides exhumation processes^51^.

In terms of geomorphology, the above-described geological contrast is well expressed in the landscape of both domains. For the internal domain, rump-like landforms define the “Croupes” morphstructural unit of the Beni Said massif^52^ (Fig. 1). It is dominated by elongated hills, cut short near the shoreline by subvertical normal faults. To the West, N-S oriented carbonate rock ridges separate the Croupes domain from that of the narrow, V-shaped valleys of the External Domain. The latter valleys are all regrouped into the “Sillons d’Erosion Differentielle” morphostructural unit : an elongated, N-S oriented tectonic window^52^ or Differential Erosion Furrows (DEF) in English. It is dominated by furrow-like valleys generated by differential erosion processes that exploit the lithological contrast between the fine-grained sedimentary shales and marls of Tangier unit and the harder more compact limestones and flysch formations of the “Dorsale Calcaire” and “Nappes de Flysch” subdomains respectively (Fig. 1). The latter units constitute the lateral boundaries of these deep valleys and occupy higher altitudes compared to the underlying Tangier formations.

Interestingly, the administrative boundary of Tetouan province includes both the Croupes and the DEFs morpho structural units. This is due to the complex hydrographic network evolution that separated the Martil drainage basin from the Beni Said Massif. Such structure can induce large differences in terms of lithological and structural conditions between the three study areas.

## Methodology

### Research strategy

**Fig. 2:**
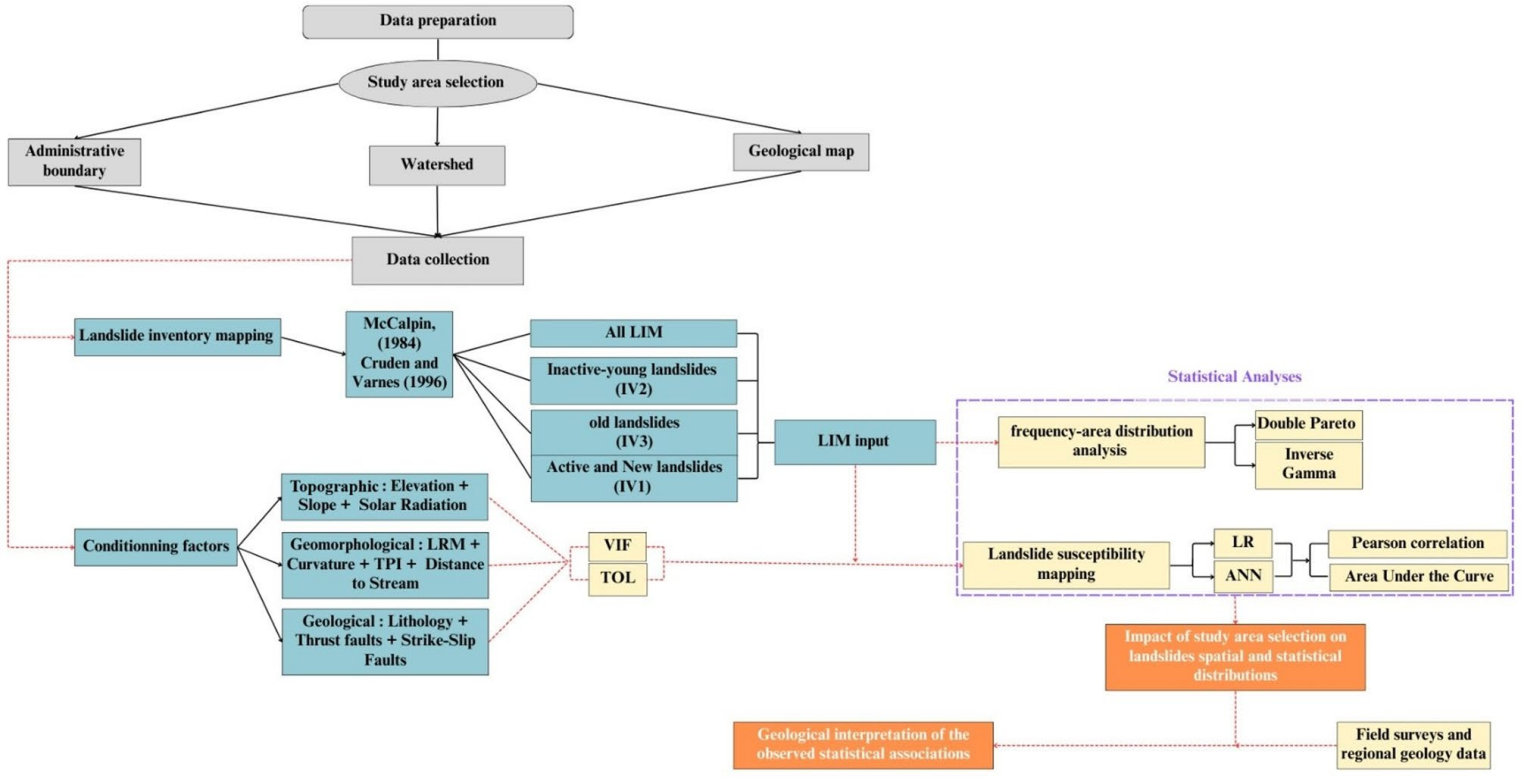
Flowchart of the study methodology. VIF: Variance Inflation Factor, TOL: Tolerance, LR: Logistic Regression, ANN: Artificial Neural Network, LRM: Local Relief Model, TPI: Topographic Position Index, LIM: Landslide Inventory Mapping.

This study aims to analyze the impact of two key parameters on the performance of landslide susceptibility models: (i) the influence of study area selection and (ii) the effect of integrating multi-generational landslides inventory maps. To achieve this, a methodological approach combining several techniques was adopted. The analysis of study area influence was conducted by considering three distinct boundaries: the administrative boundary of the province of Tétouan, the 1:50000 geological and topographic map of Tetouan, and the Oued Martil watershed named Tetouan watershed in this paper for simplification purposes. The three study areas partially or totally overlap and are mostly located in a geologically, geomorphologically, and climatically homogeneous settings at the exception of the latter boundary that is partially made of different morpho-tectonic units (Fig. 2). As such, our methodology is based on landslide inventory mapping, frequency-area distribution analysis, assessment of conditioning factors, susceptibility modeling, and validation of the results (Fig. 2). The interpretation of the results relies on geological and geomorphological controls specific to the study area so as to assess the impact of their spatial distribution on the output models. Furthermore, the influence of multi-generational landslides inventory data was examined by integrating three distinct temporal classes: recent active landslides that occurred before 2003 (IV1), Inactive-young landslides (IV2), and old landslides (IV3). These data were tested across the above-described boundaries in order to assess the influence of a landslide’s age-activity category in different spatial boundaries (Fig. 2). Landslides that occurred after 2003 were used for validation as explained in Sect. 3.6.

###  LIM preparation

In this poorly documented area regarding landslides, the experience of the co-authors of this article has been essential, as they have extensive experience in preparing LSMs in the geomorphological setting of Northern Morocco^27,35,38,54-56^. Additionally, several data collection techniques have been implemented, including field surveys, analysis of remotely sensed data, and interviews with local authorities and residents of the study area. Among the remotely sensed data, a collection of stereoscopic aerial photographs from 2016 to 2022 were provided by the *Centre Royal de Télédetection Spatial* (https://www.crts.gov.ma/), with respective spatial resolutions of 1:300,000, 1:200,000, and 1:700. Furthermore, 1:50,000 official topographic maps produced in the 1970s, newspaper articles, previously published theses^54,57^, and field surveys conducted from 2017 to 2022, helped to complete and update the LIM (Table 1). Although automatic LIM tools are considered valid options for identifying landslides^58–61^, expert-based landslides inventorying using aerial photography is a more reliable approach in complex geomorphological settings where multigenerational mass movements overlap and generate complex landforms that automatic tools cannot accurately identify^62^. To discriminate between the inventoried landslides, the typology of each mass movement was determined using the^63^ classification system. This data allowed comparing landslides processes characterizing the external domain to those occurring in the internal domain.

**Table 1:** Database used for Preparing LIM used in this study.

Data	Spatial extension	Temporal coverage
Aerial photograph sets	Entirety of the study area	1955–1958 – 1965–1984 – 2010
Ortho-images	Entirety of the study area	2009–2017 – 2018–2019
Multidate Google-earth images	Entirety of the study area	2003-2006-2009-2010 to 2020.
1:50,000 topographic maps	Entirety of the study area	Late1960’s to early 1970’s
1 :25,000 topographic maps	Entirety of the study area	2009–2010
Detailed topographic sketches and associated aerial photographs (1:7000).	Territory of urban and rural centres	Varies depending on the location.
1 :50,000 geological maps	Entirety of the study area (at the exception of Larbaa Ayacha and Souk Elkola maps which were completed in this study)	-
Digitial Elevation Model (5 m)	Entirety of the study area	2009–2010
High resolution (15 cm) aerial photographs	Oued laou and Beni Said communes	2017
Medium resolution (30 cm) aerial photographs	Zinat and Amsa commune	2014

Finally; the generated LIM dataset was divided into three groups according to the^8^ relative age classification. The first group (IV1) consists of active landslides that occurred between 2003 and 2010, as well as other processes that took place during the rainy winter of the 2017/2018 meteorological year. The second group (IV2) includes young inactive landslides that occurred before 2003, which were used to train the LSM algorithms. Finally, the third group (IV3) comprises mature inactive landslides as well as old inactive ones. To identify these landslides, multi-date earth satellite images provided by google earth were used to determine the approximate month/year of occurrence.

### Frequency area distribution (FAD) analysis

As demonstrated in previous research, FAD analysis constitutes a crucial step that allows characterizing the size distribution of the inventoried landslides as well as the completeness of the LIM as a whole^38,64- 66^. Generally speaking, a quasi-complete landslide database is expected to fit both the Double Pareto and the Inverse Gamma theoretical models as shown by^67,68^ respectively. Failure to adjust to either of the models, especially in the rollover area, indicates incomplete datasets that lack significant representation of mid-size and/or small landslides. The probability density function for the Double Pareto Distribution is given in Eq. 1 while that of inverse gamma is presented in Eq. 2.

1 $$\:\boldsymbol{f}\left(\boldsymbol{x}\right)=\frac{1}{\boldsymbol{\beta\:}\boldsymbol{\Gamma\:}\left(\boldsymbol{\rho\:}\right)}{\left(\frac{\boldsymbol{\beta\:}}{\boldsymbol{x}-\boldsymbol{\gamma\:}}\right)}^{\boldsymbol{\rho\:}+1}\boldsymbol{e}\boldsymbol{x}\boldsymbol{p}\left(\frac{\boldsymbol{\beta\:}}{\boldsymbol{x}-\boldsymbol{\gamma\:}}\right)\:;\:\boldsymbol{\Gamma\:}\left(\boldsymbol{\upalpha\:}\right)={\int\:}_{0}^{\mathbf{\infty\:}}{\mathbf{t}}^{\boldsymbol{\upalpha\:}-1}{\mathbf{e}}^{-\mathbf{t}}\mathbf{d}\mathbf{t}\:\:(\boldsymbol{\upalpha\:}>0)$$

Where* p* is a parameter of the gamma function, (α > 0), β a continuous scale parameter (β > 0), γ is a continuous location parameter and Γ is the gamma function.

2 $$\:\boldsymbol{f}\left(\boldsymbol{x}\right)=\frac{\boldsymbol{\beta\:}}{{\boldsymbol{A}}_{\boldsymbol{p}}}\:\frac{{\left[1+{\left(\frac{{\boldsymbol{A}}_{\boldsymbol{m}\boldsymbol{a}\boldsymbol{x}}}{{\boldsymbol{A}}_{\boldsymbol{p}}}\right)}^{-\boldsymbol{\alpha\:}}\right]}^{\frac{\boldsymbol{\beta\:}}{\boldsymbol{\alpha\:}}}}{{\left[1+{\left(\frac{\boldsymbol{x}}{{\boldsymbol{A}}_{\boldsymbol{p}}}\right)}^{-\boldsymbol{\alpha\:}}\right]}^{1+\frac{\boldsymbol{\beta\:}}{\boldsymbol{\alpha\:}}}}\times\:{\left(\frac{\boldsymbol{x}}{{\boldsymbol{A}}_{\boldsymbol{p}}}\right)}^{-\boldsymbol{\alpha\:}-1}$$

Where x is the landslide area, A_max_ is the largest landslide area in the data; A_p_ is the rollover location; α and β are respectively the positive and negative exponents of power law functions.

In this study we tested the completeness of our LIM based on the above models. To achieve this, FAD curves were generated for all three investigated territories using a completely automated FAD fitting tool^69^. First the algorithm was fed LIM data that excluded relict processes and included Inactive-young landslides (IV2). The second test was done using only inactive-mature and Inactive-young processes (IV3) and the third was conducted using exclusively new/active mass movements (IV1). This allows to compare the distribution of three types of landslides that occurred under different climatic and geological conditions. As a results, one can understand the effect of study area selection and typological discrimination on FAD parameters estimation. Given that the latter are tied to the evolution of the landscape as a whole^38,70,71^, this information allows for a comprehensive training data preparation and filtration based on statistical indexes and numerical estimations.

### Landslides conditioning factors

To investigate the impact of study area selection on the statistical correlation between landslides and their conditioning factors, and to select only the most significant factors/variables contributing to instability processes for each landslide category (active, ancient and relict), we conducted a multicollinearity analysis through calculating the tolerance coefficient for each variable. This method is commonly used to assess the correlation between two or more predictor variables in a multiple regression model^72^. Multicollinearity refers to the non-independence of landslide conditioning factors, which can arise in datasets due to their high linear correlation^73^. This approach was deemed useful in numerous LSM studies (e.g^74,75^. The formula for calculating the tolerance coefficient (TOL) for each landslide conditioning factor is given by Eq. 3:

3 $$Tol = 1 - R_{J}^{2}$$

where R_j_² is obtained through regressing the independent variable in question onto the remaining independent variables used in the LR analysis^76^. If the value of the calculated tolerance coefficient (Tol) is less than 0.1, this indicates multicollinearity, and consequently the corresponding variable should be excluded from the landslides predictive model.

###  LSM computation

In this work, the establishment of LSMs aims to not only assess the landslides spatial hazard in Tetouan province, but rather to study the impact of study area selection (administrative boundary, geological map and watershed) on the spatial distribution of different landslides processes. In this respect, the choice of the LSM computation technique was based primarily on their reliability when faced with significant input data variation, as well as their effectiveness and overall popularity so as to maximize the replicability, comparability and generalizability of the results. For these reasons, our extensive literature review revealed that logistic regression (LR) technique and artificial neural networks (ANNs) constitute the best modelling techniques for our purpose since they revealed a high effectiveness over the last two decades^20,26-28,77-79^.

In the case of Artificial neural networks (ANNs), it is an artificial intelligence method widely used in LSM mapping studies^79–81^. These algorithms were recognized as robust computational tools capable of acquiring, representing and analyzing multivariate datasets^82^. They offer a number of advantages over conventional statistical methods. In fact, ANN algorithms are independent of the statistical distribution of the data, which means that they require no pre-processing steps^83,84^. The mathematical model used to calculate landslides spatial probability according to this technique is given by Eqs. 4 and 5.

4 $$Net = \mathop \sum \limits_{{i = 1}}^{n} x_{i} w_{i}$$

5 $$\:LSI=\mathrm{P}\mathrm{j}\:=\:\frac{1}{1+{e}^{-Net}}$$

where P_j_ is the pseudo-probability for each individual cell in the study area, and net is the input received by each neuron in the hidden layers. A more detail explanation of how the “net” value is calculated is given by^79^.

The second technique is Logistic Regression (LR) method, which is a useful model for predicting binary outcomes based on a set of predictors. It can be used in landslides research to determine the relationship between their spatial occurrence and their potential conditioning factors. Currently, it is the most popular multivariate method in LSM^85^. To implement this algorithm, the dependent variable must be dichotomous and the explanatory variables must exhibit little or no multicollinearity. Next, the conditional probability of landslides occurrence P (y = 1|x) is calculated using Eq. 6 and Eq. 7.

6 $$\:\mathrm{P}\:(\mathrm{y}\:=\:1\:|\mathrm{x})=\frac{1}{1\:+\:{e}^{-z}}$$

7 $$z{\text{ }} = {\text{ }}b_{0} + {\text{ }}b_{1} x_{1} + {\text{ }}b_{2} x_{2} + \cdots + {\text{ }}b_{n} x_{n}$$

where b_0_ is the intercept, x_1_, x_2_ and x_n_ are the independent variables, and b_i_, b_2_ and bn are their coefficients.

### Validation dataset

**Fig. 3:**
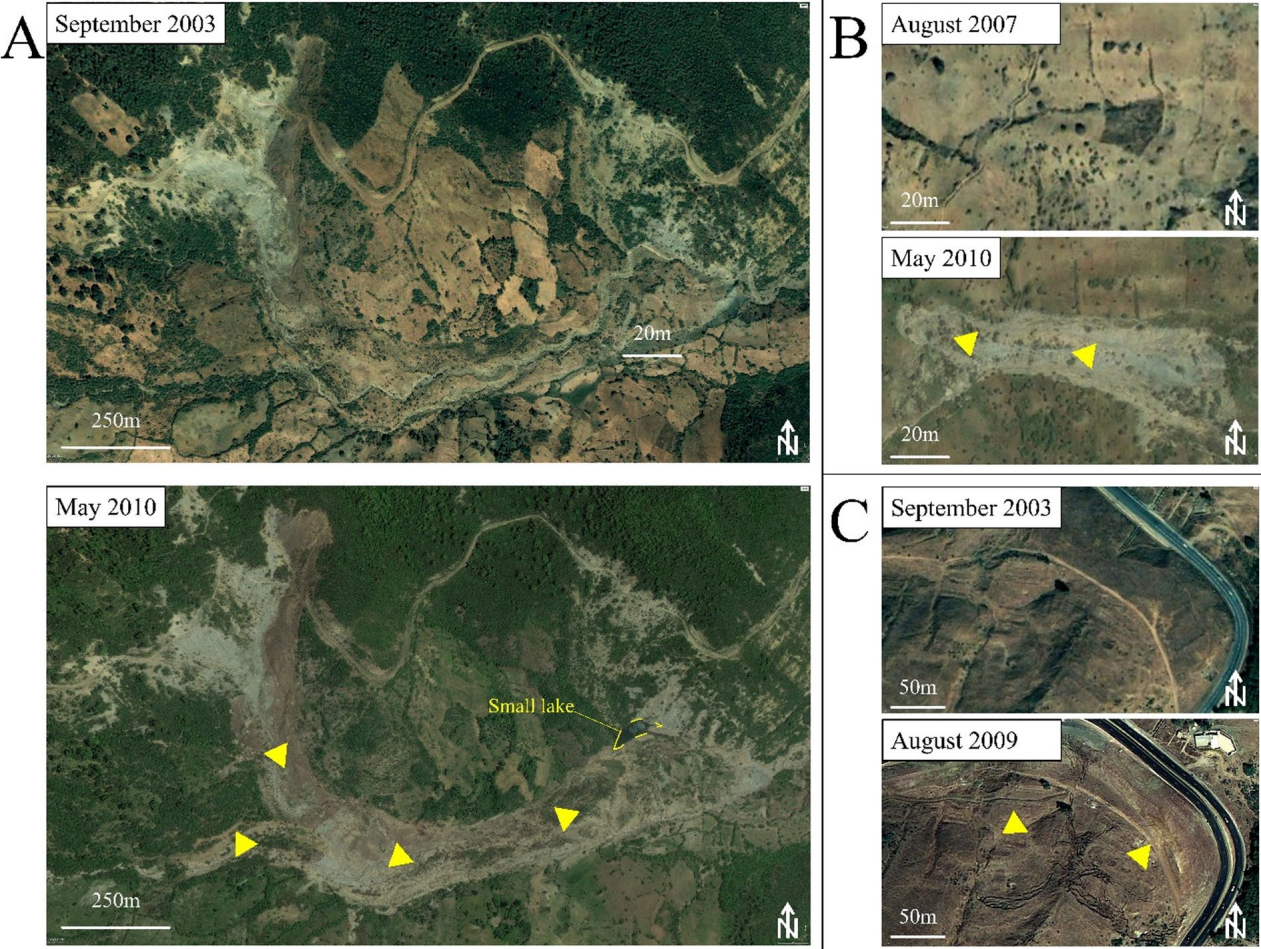
(**A**) Reactivated slide-earth flow near Beni-Idder (X= -5.544°; Y = 35.382°). (**B**) Earth flow triggered in 2009 (X= -5.362°; Y = 35.373°). (**C**) Complex landslide reactivated in 2009 (X= -5.587°: Y = 35.565°) (Hight resolution images extracted from *google earth Pro*, https://earth.google.com/web/).

Preparing the validation dataset is an important step in any LSM investigations. Conventionally, the LIM is randomly split into two groups, one comprised of 70% of the original data and the other containing only 30%^26,86^. However, this strategy only makes sense if the current and past slope dynamics are controlled by the same conditioning and triggering factors. In fact, most areas of the world were impacted by major climate change and baselevel fall events since the early Quaternary times^87,88^, leaving behind large-scale relict landforms in general, and landslides in particular, that do not necessarily reflect the current geodynamics. Therefore, the validation dataset may contain such processes and thus produce false accuracy assessments that do not reflect the predictive power of the LSM but its goodness of fit to the data. To avoid this situation, we used the threshold year of 2003 to split our LIM to training and validation data. This year was chosen because google earth coverage of our study area started in September 2003, thus providing the ability to date all processes that occurred afterwards. Examples are given in Fig. 3, where some Inactive-young processes (IV2) were reactivated and some new ones were triggered during the exceptionally rainy period of 2003–2009 (IV1). Accordingly, a validation dataset was generated, which is exclusively composed of new landslides processes that characterize the current slope dynamics of the study area. This information allows the assessment of the true predictive power of the computed LSMs given that the training data LIM includes only mass movements that occurred before 2003 (IV2).

### LSM validation using area under the curve (AUC) technique

The Area Under the Curve technique is used to quantitatively assess the predictive power of LSMs^36,89,90^. It is based on calculating the Area under the Receiver Operating Curve (ROC), which is constructed through plotting sensitivity (true positive rate) values against specificity (true negative rates)^91^. These values can be calculated using the following Eqs. 8 and 9.

8 $$\:Sensivity=\:\frac{TP}{TP+FN}$$

And.

9 $$\:Speccificity=\:\frac{TN}{TN+FP}$$

Where, TP is the number landslides pixels that were correctly classified, TN is the number of non-landslides pixels that were correctly classified, FP is the number of landslides pixels falsely classified by the model and FN is the number of landslides pixels falsely classified by the model.

Depending on the AUC analysis results, a LSM can be classified as bad (< 0.5), poor (0.5–0.6); average (0.6–0.7), good (0.7–0.8), very good (0.8–0.9) or excellent (> 0.9)^92,93^. However, as stated before, we interpret AUC values in this study as goodness of fit assessment to landslides mostly triggered by the 2003–2009 rainy period.

## Results and discussion

### LIM distribution

**Fig. 4:**
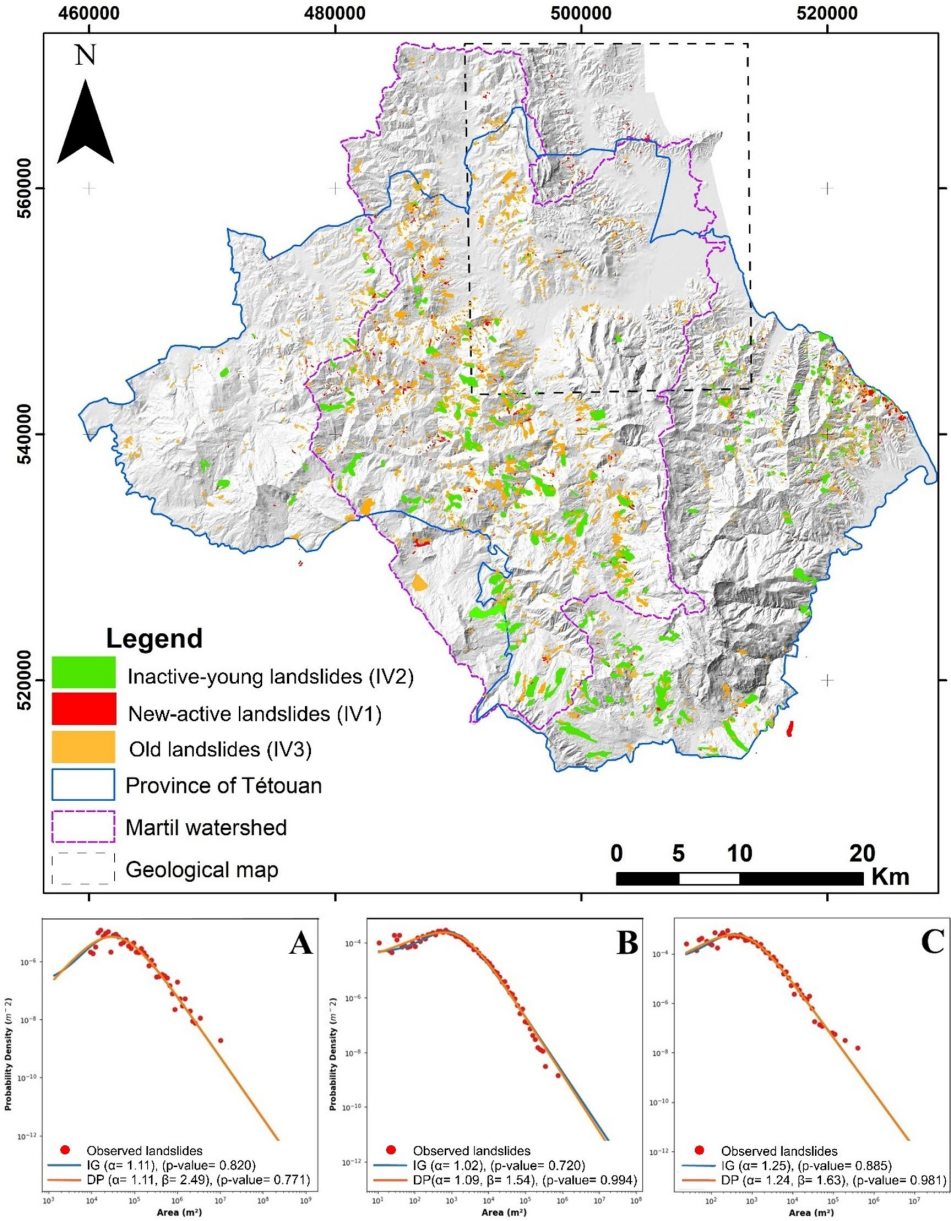
Landslide inventory map and results of FAD analyses. (**A**) FAD curve obtained using Relict landslides (IV3). (**B**)- FAD curve obtained using old landslides (IV2). (**C**) FAD curve obtained using new and active landslides (IV1), (used software: *ArcGIS Pro 3.5*, https://pro.arcgis.com/fr/pro-app/).

The LIM produced in the study area includes a total of 5,208 landslides, comprising 3,716 inactive-young landslides (IV2), 367 relict processes (IV3), and 1,125 new and active mass movements (IV1). As stated before, the latter category is comprised of landslides triggered by the exceptionally rainy period of 2003–2010. With regards to LIM completeness, the FAD analyses performed on all three categories reveal the completeness of the data since the output FAD curves fit well the Double-Pareto and Inverse-Gamma models, with p-value for the Kolmogorov-Smirnov test largely exceeding the 0.05 threshold. The contrast in terms of size distribution between the above landslides categories is so clear in the FAD scaling and positioning parameters (Fig. 4). In fact, the relict landslides (IV3) yield the highest scale parameter (β-value = 2.49) compared to both the new-active (IV1) and Inactive-young landslides (IV2) (Fig. 4A). This indicates that relict landslides are significantly larger especially given the higher positioning parameter value of this category (ten times that of IV- 2 and IV-1). As for the other two categories, the comparison of FAD results reveals a mostly similar distribution both in terms of scaling (β-value = 1.54 and 1.63 for IV2 and IV1 respectively) and positioning parameters (Fig. 4B, C). However, the large-scale landslides of the new/active LIM data seem to be under-represented compared to the Inactive-young LIM (IV2) curve (Fig. 4B, C).

To sum up the results of this analysis, our findings definitively reveal the difference between the β-value of relict processes compared to other landslides, which translates a significant difference between current and old quaternary times in terms of geological, geomorphological, hydrological and climate conditions. Such findings are similar to previous research that proved the effectiveness of FAD parameters in revealing the contrast between landslides conditioning and triggering factors in diverse landscapes^38,64,70,71^, 94]–^97^.

### Landslides conditioning factors

Of the various landslides conditioning factors used to build the LSMs in this study, no factor is associated with low tolerance values. Thus, they are found to be significant for the three investigated sectors and all the LIM datasets. However, it is not the multicollinearity of the explanatory variables that is in question. It is in fact the sensitivity of tolerance values to changes in the spatial extent of the study area. In this respect, most conditioning factors show no difference between the watershed, Tetouan map and Tetouan province is observed (Table 2). An exception to this rule is elevation, which exhibits tolerance values of 0.79 to 0.78 for all three investigated sectors without any notable variability between the three LIMs. Nevertheless, the tolerance values drop significantly: around 20% for the Tetouan map model and 30% for Tetouan province (Table 2). The second variable that can be distinguished from the bunch is solar radiation (SR), which is mostly the same for the watershed and the Tetouan map models and completely different in the Tetouan province one, with VIF of the latter being 20% lower than the other two. This is normal given the different slope aspects of the Internal Rif domain, which is not covered by the Tetouan map and watershed models (Table 2). The same observation can be made regarding the slope degree variable with the same geomorphological explanation for the results. Finally, the distance to thrust fault variable shows a similar difference between the investigated sectors, with tolerance values for the watershed and province models largely surpassing those of the Tetouan map model. This can be explained by the absence of thrust faults in the Tetouan map, which mostly is mostly made of lower slope, post-nappe formations (Fig. 1).

**Table 2:** Multicollinearity assessment test using tolerance values.

	Watershed			Map			Province		
	ALL_LIM	IV3	IV2	ALL_LIM	IV3	IV2	ALL_LIM	IV3	IV2
Lithology	0,95	0,98	0,95	0,85	0,87	0,85	0,84	0,83	0,85
LRM	0,58	0,54	0,58	0,61	0,61	0,61	0,69	0,67	0,69
**Elevation**	**0**,**79**	**0**,**71**	**0**,**78**	**0**,**57**	**0**,**59**	**0**,**59**	**0**,**42**	**0**,**45**	**0**,**43**
**Slope**	**0**,**76**	**0**,**76**	**0**,**76**	**0**,**59**	**0**,**56**	**0**,**59**	**0**,**63**	**0**,**62**	**0**,**63**
**SR**	**0**,**83**	**0**,**83**	**0**,**83**	**0**,**82**	**0**,**82**	**0**,**82**	**0**,**65**	**0**,**68**	**0**,**66**
Dis. SSF	0,83	0,83	0,82	0,73	0,72	0,73	0,82	0,87	0,83
**Dis. THF**	**0**,**83**	**0**,**84**	**0**,**83**	**0**,**66**	**0**,**67**	**0**,**66**	**0**,**93**	**0**,**92**	**0**,**92**
TPI	0,70	0,70	0,71	0,62	0,64	0,62	0,49	0,50	0,48
Curvature	0,50	0,51	0,51	0,46	0,45	0,46	0,56	0,58	0,55
Dis. Streams	0,84	0,84	0,85	0,90	0,89	0,90	0,92	0,95	0,93

###  Landslides susceptibility maps (LSMs)

#### Cartographically delimited study area: Tetouan map LSMs

**Fig. 5:**
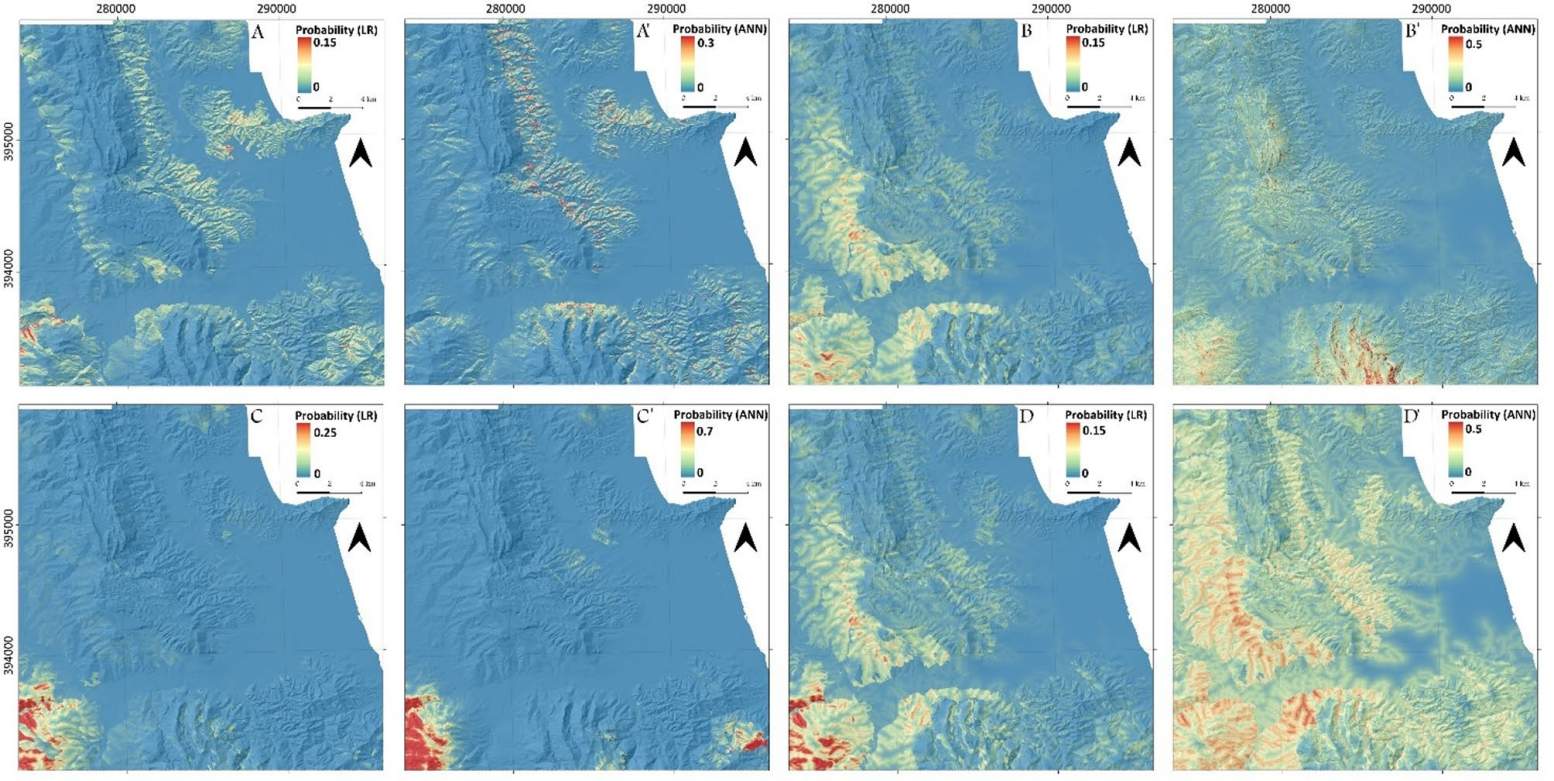
Output LSMs for Tetouan map study area. (**A**) and (**A’**) LR and ANN LSMs respectively using IV-1. (**B**) and (**B’**) LR and ANN LSMs respectively using IV-2. (**C**) and (**C’**) LR and ANN LSMs respectively using IV-3. (**D**) and (**D’**) LR and ANN LSMs respectively using all data LIM, (used software : *ArcGIS Pro 3.5*, https://pro.arcgis.com/fr/pro-app/).

The LSMs obtained for this study show that for Tetouan map, the new/active LSMs are the most conservative with medium and high susceptibility values covering a very small portion of the study area (Fig. 5A and A’). In addition, the spatial distribution of such moderate to high susceptible values is mainly correlated with narrow valleys. This shows that new and active processes are mostly shallow and small-scale landslides that are tied to surface runoff erosion. With regard to the impact of the LSM computation techniques on the output model, no significant difference is observed between the LR (Fig. 5A) and the ANN LSMs (Fig. 5A’). Conversely, the old LIM produces a large difference with regard to the spatial distribution of the LR (Fig. 5B) and ANN LSM (Fig. 5B’). Moreover, this spatial distribution is largely different from that of the first two LSMs (Fig. 4A and A’) with medium to high susceptibility values being mostly tied to thrust faults for the LR model and the *Dorsale Calcaire* morphostructural unit in the ANN model. As for the relict data LSMs, they also exhibit a completely different distribution (Fig. 5C and C’), which highlights the contrast between new/active, old and relict processes in the study area. In fact, the high susceptibility values are concentrated in relatively high elevation sectors of the study area especially in the SW of Tetouan map (Fig. 5 -C and -C’). The territory corresponding to the hilly topography of the *Dorsale Calcaire* tectonic unit shows low to very low susceptibility values. Finally, the LSMs produced using all landslides inventoried within the perimeter of Tetouan map are characterized by a very liberal distribution especially compared to the other three LSMs. (Fig. 5D, D’). This is true for both the LR and ANN models. However, both techniques tend to produce different spatial distributions, which is comparable to the difference observed in old landslides LSMs of Fig. 5 -B and -B’. Also, these LSMs are clearly influenced by all processes since the input data is basically a combination of all previous three.

#### Geomorphologically delimited study area: Martil watershed LSMs

**Fig. 6:**
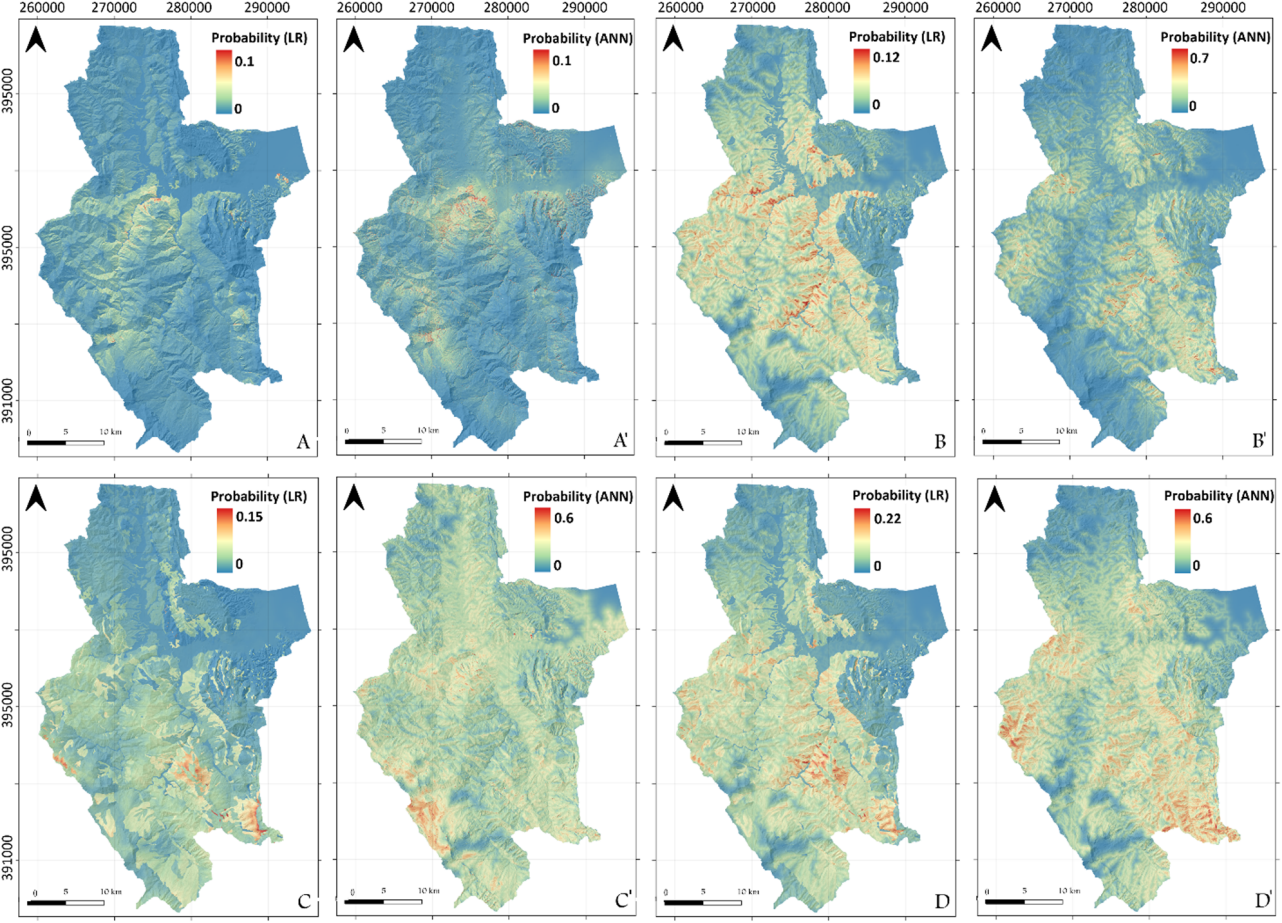
Output LSMs for Tetouan map study area. (**A**) and (**A’**) LR and ANN LSMs respectively using new active LIM. (**B**) and (**B’**) LR and ANN LSMs respectively using old LIM. (**C**) and (**C’**) LR and ANN LSMs respectively using relict LIM. (**D**) and (**D’**) LR and ANN LSMs respectively using all data LIM, (used software : *ArcGIS Pro 3.5*, https://pro.arcgis.com/fr/pro-app/).

Based on the visual interpretation of the results, one can confirm that the same tendencies observed in the Tetouan map LSMs are mostly repeated in the Martil watershed study area. For instance, the new and active processes also produce the most conservative model of the bunch (Fig. 6A, A’). Also, their spatial distribution is mostly controlled by gullying and surface runoff erosion (Fig. 6A, A’). However, the ANN model (Fig. 6A’) is found to be slightly more liberal than the LR one (Fig. 6A). As for the second dataset, both the LR and ANN models present mostly similar distributions that are influenced by the geological structure of the study area, with the LR LSM being the more liberal in this case (Fig. 6B). This demonstrates the impact of more data entries on ANN models (Fig. 6 -B’) that are known for their sensitivity to input data variation. Furthermore, since the Tetouan map ANN models for the same landslide types are clearly different, but those covering Martil watershed are mostly the same, the above-mentioned effect is made further proven. Nevertheless, LSMs that are based on relict landslides inventory (Fig. 6C, C’) are significantly different where the LR model is clearly dependent on the lithology of the study area (high susceptibility = slope deposits terrain) (Fig. 6C), while the ANN one reveals a clear dependency on elevation (high susceptibility = high elevation areas) (Fig. 6C’). As for the last dataset (i.e. old landslides), the results are similar to Tetouan map since the output LSMs are simply a combination of all other LSMs (Fig. 6D, D’).

#### Models based on administrative boundaries: Tetouan Province LSMs

**Fig. 7:**
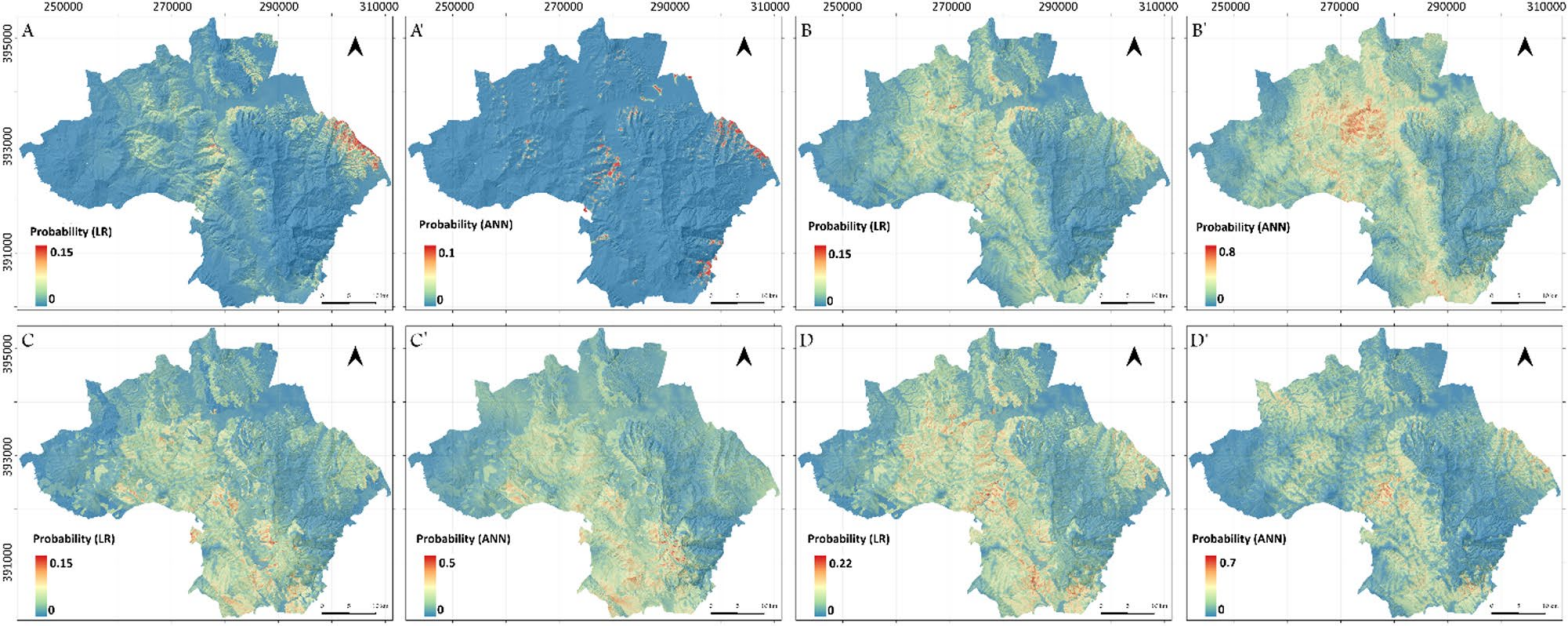
Output LSMs for Tetouan province study area. (**A**) and (**A’**) LR and ANN LSMs respectively using new active LIM. (**B**) and (**B’**) LR and ANN LSMs respectively using old LIM. (**C**) and (**C’**) LR and ANN LSMs respectively using relict LIM. (**D**) and (**D’**) LR and ANN LSMs respectively using all data LIM, (used software : *ArcGIS Pro 3.5*, https://pro.arcgis.com/fr/pro-app/).

Comparing the LSM results for Tetouan province to the previously shown LSMs is very important from a geomorphological stand point since this study area corresponds to an administrative district that involves two largely contrasted morpho-structural and geological domains (The external and internal domains). Knowing this, one can clearly see the difference in current slope dynamics manifested in the new and active LSM models (Fig. 7A, A’). In fact, the high susceptibility areas are spatially correlated with gullies in the external domain (similar to Tetouan map and Watershed LSMs), but seem to correspond to coastal areas in the Croupes morphostructural unit (Fig. 7A and A’). The latter results are supported by recent studies that focused on the recent and old coastal slope dynamics of the Croupes unit^14^. With respect to the old landslides LSM, the spatial distribution of landslides susceptibility calculated using LR algorithm (Fig. 7B) did not change for the DEF unit compared to Tetouan map and watershed models (Fig. 7A and C respectively). However, the ANN LSM is clearly more liberal in the External Domain of the Tetouan province model (Fig. 7B’). This is due to the fact that ANN models are very sensitive to input variation. In the Croupes domain the results are contrasted with the new active LSMs distribution (Fig. 7A, A’), since the coastal areas are characterized by low to medium probability values. In the case of relict LSMs, the Tetouan province models are very similar to the Tetouan watershed models, with lithology and distance to thrust fault being the most relevant factors in the external domain for LR LSMs (Fig. 7C, C’). This proves that changing study area boundaries in similar geomorphological and/or geological conditions is not translated to significant differences in the output model. However, elevation does not seem to control the ANN models as much for Tetouan province (Fig. 7C’). For the *Croupes* domain, no high probability areas are shown (Fig. 7C, C’), which does not correspond to results presented by the authors mentioned previously in this section. This proves how the contrasted geomorphological setting between the External and Internal Rif domains impacts the outcome of the modelling when both geological domains are included in the same study area. The final models (Fig. 7D, D’) are simply a combination of all previous three as is the case of other study areas, with ANN being the most conservative in this case.

### Impact of study area selection on LSM predictive power

**Fig. 8:**
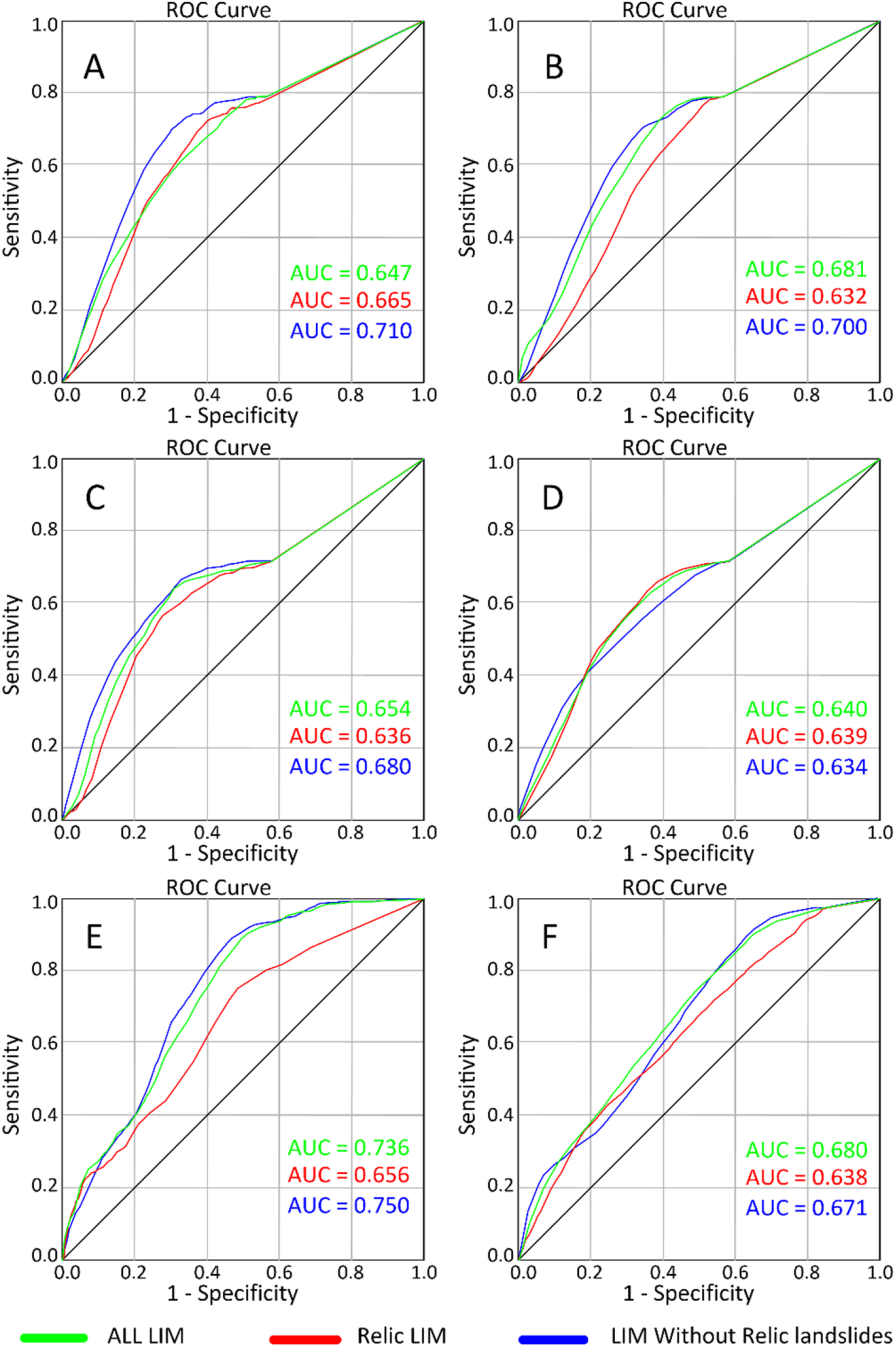
LSM validation results using AUC technique. (**A**) Tetouan watershed LSMs using LR. (**B**) Tetouan watershed LSMs using ANN. (**C**) Tetouan province LSMs using LR. (**D**) Tetouan province LSMs using ANN. (**E**) Tetouan map LSMs using LR. (**F**) Tetouan map LSMs using ANN, (used software : *ArcGIS Pro 3.5*, https://pro.arcgis.com/fr/pro-app/).

As stated, before in the methodology and introduction sections, our main objective is to test the effect of study area selection on LSM distribution and not to test the accuracy of these models. Therefore, the AUC analyses presented in Fig. 8 must be interpreted as a goodness of fit test to compare the distribution of the newly triggered landslides associated to the exceptionally wet period of 2003–2009, to that of Inactive young and relict processes (IV2 and IV3). With this in mind, two main observations remain consistent in the results: (i) both LR and ANN techniques produce the same output using the same input LIM and (ii) the relict LSM models produce the least accurate output with the exception of the ANN LSM for Tetouan province study area (Fig. 8D). In general, the AUC values obtained for all models can be deemed average to acceptable for Tetouan Watershed and Tetouan province models, and good for the Tetouan map LSM (Fig. 8). This can be explained by the fact that new and active landslides are very different from Inactive-young and relict ones. Therefore, the latter cannot be used to predict the spatial probability of the former. In addition, the relatively high AUC values for Tetouan map can be interpreted by the absence of Inactive young and relict processes in Tetouan map territory due to the maturity of the relief. As for the relict processes, the low AUC values are indicative of the lack of spatial correlation between the newer and shallower landslides compared to the deep and relict ones that are mostly linked to interglacial quaternary periods (Fig. 8).

### Spatial correlation between relict, old and new landslides

To further assess the spatial correlation between old and new processes, we used the Pearson correlation coefficient was calculated to compare new/active LSMs to other models (Table 3). The first reliable observation across all techniques and study areas is the fact that ANN models are characterized by lower correlation compared to LR LSMs (Table 3). With regard to input data impact on LSM correlations, relict LSMs seem to be the least similar to new active LSMs for both LR and ANN techniques and across all three study areas (Table 3). This further confirms the large contrast between relict processes and the new and active ones in the study area. A fact that was previously discussed by^27^ in Ain Lahcen commune. As for the study area and its influence on LSMs correlation, the Tetouan watershed LSMs for Inactive-young and relict landsides tend to produce slightly higher Pearson coefficient values for both LR and ANN algorithms. However, the difference is more relevant in the case of Tetouan province and Tetouan watershed LSMs, with the former corresponding to the lowest correlation spatial values. This further exposes the impact of the geological and geomorphological settings of the external and internal Rif domains on the coherence of the LSM models, which was revealed in both previous paragraphs.

**Table 3:** Pearson correlation coefficient between the new-active distribution (LSM) and that of Inactive-young and relict processes.

Tetouan watershed			Tetouan map		Tetouan province	
	ANN IV1	LR IV1	ANN IV1	LR IV1	ANN IV1	LR IV1
ANN_all	0,298	-	0,27	-	0,102	-
ANN_WR	0,219	-	0,257	-	0,015	-
ANN_R	0,269	-	0,014	-	0,03	-
LR_all	-	0,569	-	0,38	-	0,415
LR_WR	-	0,567	-	0,251	-	0,493
LR_R	-	0,488	-	0,21	-	0,31

## Discussion

### Study area selection and LSM models

As is the case in previous studies^27,28^, the LIM preparation steps and the training data selection are proven again in this study to be of capital importance in determining the output of the analysis both in terms of spatial distribution and LSM accuracy. However, study area selection is arguably as important in LSM computation given the results of our investigations. From a spatial distribution stand point, the LSMs for Tetouan map and Martil watershed are mostly similar, while those of the Tetouan province seem to change given the introduction of a new morpho-tectonic unit. This effect was exposed in Taounate province, Northern Morocco, by^38^ who revealed the pivotal impact of landscape characteristics on LSM sensitivity and output. Other researchers working in different climate and geological settings have also demonstrated that the key to understanding landslides spatial distribution is the study area itself, which must be selected based on geomorphological basis^70,98^. In fact, geology-derived categories are known to influence LSMs, which tend to show a very high sensitivity to slight variations in either the categorization method or the geological setting itself^99^. As such, the administrative districts cannot be used as reliable study areas since they include terrain with variable and sometimes even contrasted geomorphological characteristics, such as the case of Tetouan province. Such differences do not only reflect heterogenous orographic features, but also include underlying morpho-tectonic features responsible for their occurrence. Consequently, the modelling process may fail to produce replicable results as new morphotectonic units are either added or eliminated from the study area based on purely administrative criteria.

In his famous paper^100^ talks about the two “cultures” of statistical modelling: the data modelling culture that assumes a stochastic data model; and an algorithm modeling culture that considers the experiment mostly unknown and not fully described by a pure mathematical equation. By not considering the issue of study area selection in LSM studies, one might fall into the former category where the model becomes purely data driven and may lead to irrelevant theories and questionable conclusions. This is especially true for geologists and geomorphologists alike who are not simply interested in the predictive power of the LSM itself but also use LSM analyses as tools to explain landslides spatial occurrence and correlation with potential predictive factors. Therefore, we recommend that LSM study area selection be done on geomorphological bases so as to avoid two contrasted geomorphological settings in the same model, which is the case for Tetouan province shown above and Taounate province investigated by^28,38^.

**Fig. 9:**
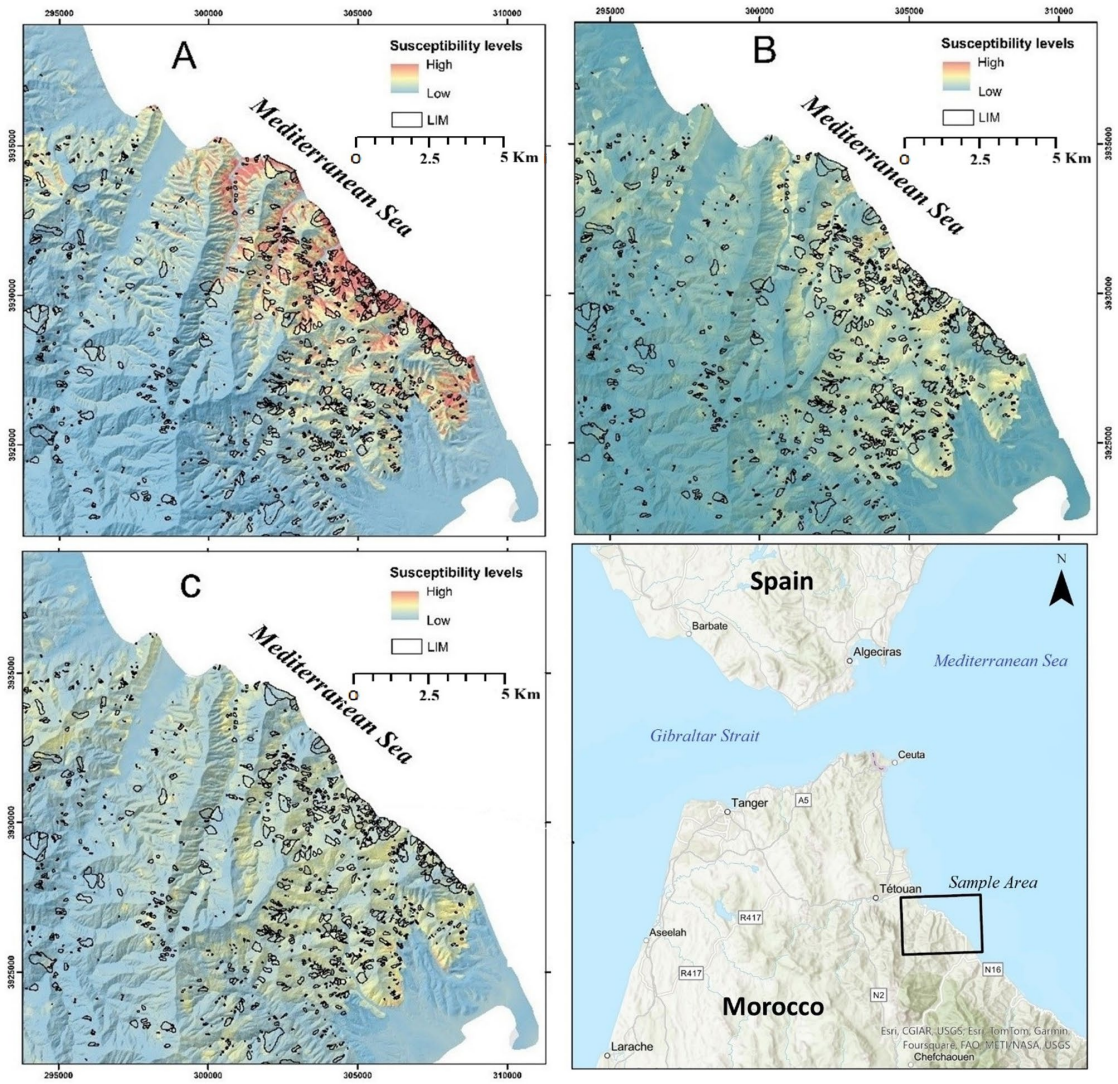
Zoomed in LSM maps from Fig.-7 showing variability in the internal domain using different inputs: (**A**) new active (IV1) LSM, (**B**) Inactive-young landsldies (IV2) LSM and (**C**) Relict landslides (IV3) LSM, (used software : *ArcGIS Pro 3.5*, https://pro.arcgis.com/fr/pro-app/).

For instance, if one focuses on the case of Tetouan province LSMs, one can clearly see that the input data variability did not affect the LSM models in the external domain (Fig. 7). Nevertheless, using different LIMs induced large sensitivity to the model for the external domain where new and active landslides produced high susceptibility values near the coastline (Fig. 9A), while relict and Inactive-young processes LSMs were characterized by low to medium susceptibility values across the entire Croupes morphostructural unit (Fig. 9B, C respectively). The LIMs used to prepare these models indicate a large density of landslides near the coastline (Fig. 9). This is also supported by site-specific research presented by^14^ in the Croupe morphological setting. To explain these observations, one must consider the impact of geological parameters (mostly distance to thrust fault and lithology) on the landslide’s spatial probability estimation. Our results suggest that in the DEF morphostructural unit, the differential erosion and hydrogeological contrast alongside thrust faults is the main conditioning factor for slope instability. However, these structures are absence in the geomorphological setting of Coupes unit, which cause an underestimation of probability given the large distance to thrust faults in this area. As for the new and active LSM (Fig. 9A), the effects of lithology and distance to thrust fault are less evident in the output LSMs (Fig. 7A, A’). Therefore, the attenuation of correlation coefficients is not as impactful in this case.

### Landslides processes in two contrasted Geomorphological settings (Internal VS external Rif)

**Fig. 10:**
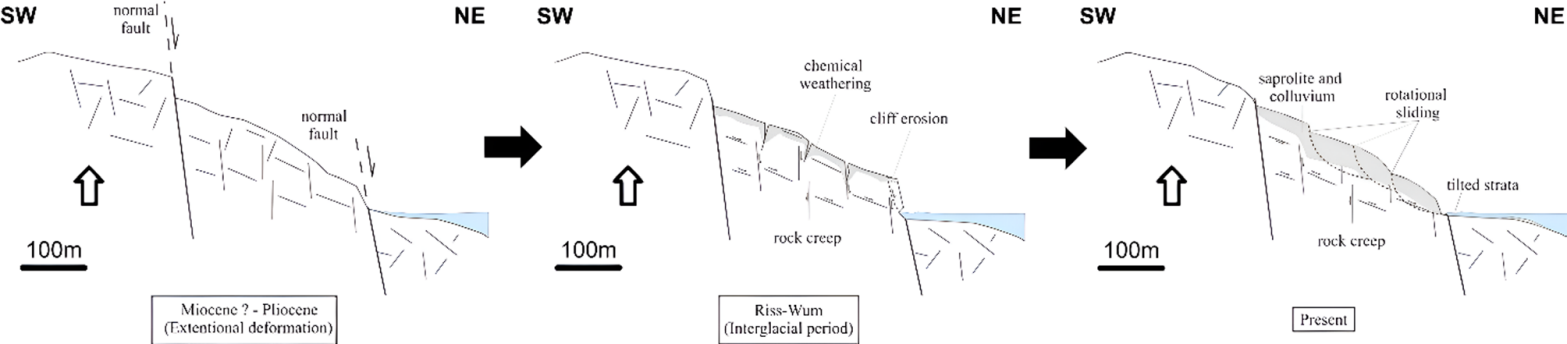
Geomorphological model describing the Quaternary evolution of landslides in the coastal environment of the internal Rif.

As shown in Fig. 7, the distribution of landslides in the internal domain is different to that of the external domain. In the former the new and active processes are mostly found alongside the coastal area. This is due to recent tectonic activity^51,101^. That controls the geometry of landslides alongside the Rif chain coastline : in Tamegaret landslide (X = -5.141°, Y = 35.493°)^14^ and SE of Chmaala town(X = -4.911°, Y = 35.309°)^51^. Also^53^, presented bivariate statistical analyses (Frequency Ratio) that prove the spatial correlation between normal faults in the internal domain and large complex landslides. Based on these results, one can resume the coastal landslides dynamics by the following model (Fig. 10) : extensive tectonic landforms sculpted by late orogenic extensive stress, which was then exacerbated by erosion and gravitational deformation. The Riss-Wurm interglacial (MIS-5) was especially rainy in the Aboran Sea region^102^, which resulted in the formation of thick saprolite and colluvium that evolved from rockcreep landforms to translational and rotational complex landslides.

**Fig. 11:**
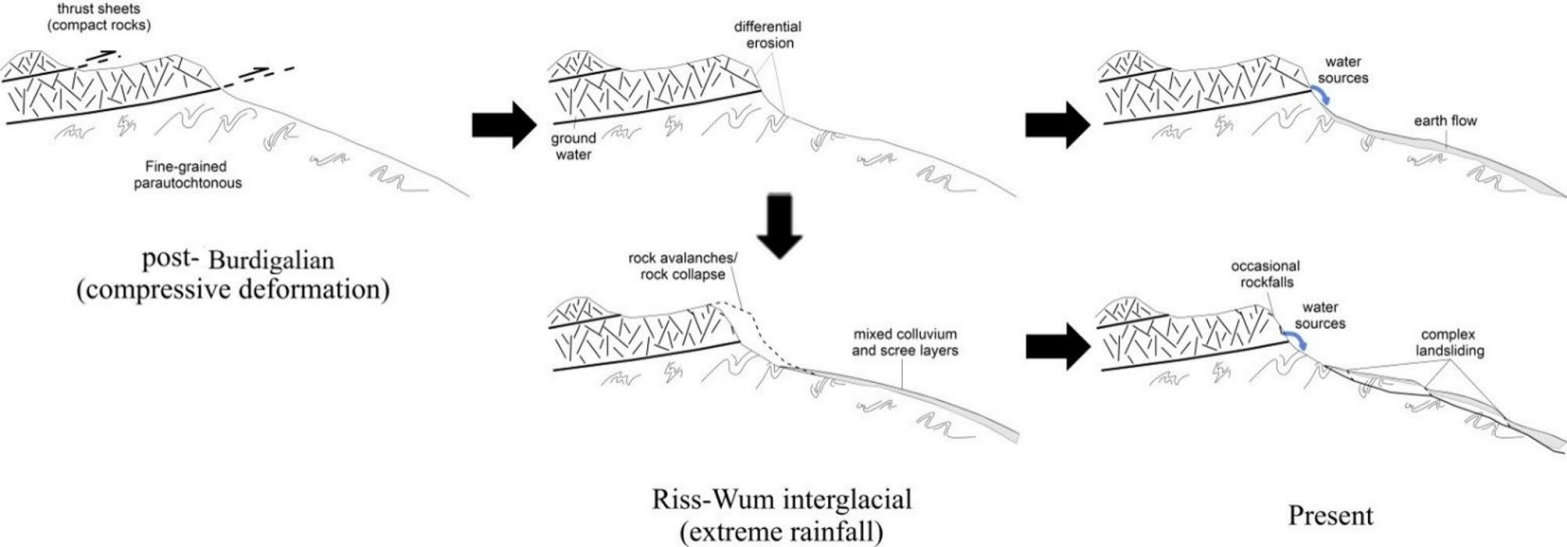
Geomorphological model describing the Quaternary evolution of landslides in the DEF morphostructural unit.

However, this dynamic is not representative of the models that cover Tetouan map and Martil watershed boundaries that use. This can be attributed to the exceptionally high concentration of landslides alongside the low-angle thrust faults of the external Rif domain, which contradicts the slope dynamics of the Internal Rif. In fact^27,53 and 56^, already presented preliminary results for the external domain that reveal high FR values near Thrust fault areas, which was confirmed by the results of our study. This is due to the hydrogeological and lithological contrasts alongside these long tectonic lineaments. As such, the slope dynamics of the external domain are mainly caused by compressive deformation (Fig. 11) unlike the internal domain where areas susceptible to landsliding correspond to extensional deformation landforms. In this case, the role of tectonic activity is indirect and thus corresponds to a drastically different spatio-statistical distribution compared to the Internal Rif. The landslides in this case are larger and originate from the multigenerational activity of scree deposits triggered by topographic growth and climate change since Villafranchien times^103^ (Fig. 11). Nowadays, the rate of this complex landsliding slowed down because of the drier climate conditions compared to those of MIS-5 and MIS-9 interglacial periods^104,105^ when most of the activity is thought to have occurred. Exceptional rain storms act however as reminders of the destructiveness of large-scale slope dynamics in the External Rif^54,106^.

### Elevation and LS values distribution using relict LIM

It is widely known that landslides are not directly affected by elevation. However, the latter can be linked to snowmelt events that release considerable quantities of water in upper and mid slopes during spring. This water infiltrates into the ground, which increases pore pressure in the subsoil layers and reduces effective normal stress on the slip surface according to Terzaghi law, thus promoting mass movements^107^. The altitude at which such phenomenon occur depends on the position of the orographic snow-line, which varies depending on latitude and season. The effect of these mechanisms was revealed in the Italian Alps for example by^108^ who demonstrated the significant impact of snowmelt events on shallow landslides especially during spring.

Nevertheless, in ice-free mountain ranges, the above-described mechanisms do not take effect. This is the case for our study area where snow fall events are very short and considered overall exceptional. For this reason, the LSMs produced in this study do not seem to suggest a high dependency on altitude at the exception of those using Relict landslides as input. Given the fact that such relict processes are all considered large-scale events^53,109,110^, their high concentration in upper to mid-slopes (which explains the positive correlation between elevation and LS values) is comparable to the findings of^111^ who showed an exceptionally high density of large-scale landslides in > 1000 m altitude areas of the Anatolian Plateau. Although in this case the spatial distribution is linked to a variation in topographic, tectonic, seismic and climate settings, we believe that the case of Martil Watershed study area is linked to a different mechanism. In fact^27^, noticed the high density of relict processes in upper slopes. Although their study focused on a relatively small area, they considered the base-level fall episodes of the last 1 million years to be the cause of the current relict landslides’ distribution. In fact, the tectonic and eustatic controls on Quaternary baselevel evolution show a 100 m sea level drop since the Pliocene^112^. Similarly, many old landforms translate the intense landslide dynamics in the near Gran Canaria island, which are related to the important river incision during the Pleistocene era^113^. In this comparable study area, three development stages were present: the first corresponds to abandoned landslides in the high slopes (0.6 My ago), the second is materialized by old and dormant landslides that mainly affected mid-slopes (Middle to upper Pleistocene) and finally Holocene landslides that are still active until this day and seem to be affected by the current runoff erosion. Although radiometric dating of landslides in the Rif chain is not available hitherto, the current U-shaped morphology of the Differential Erosion Furrow morphstructural unit^52^ is most likely linked to similar dynamics.

**Fig. 12:**
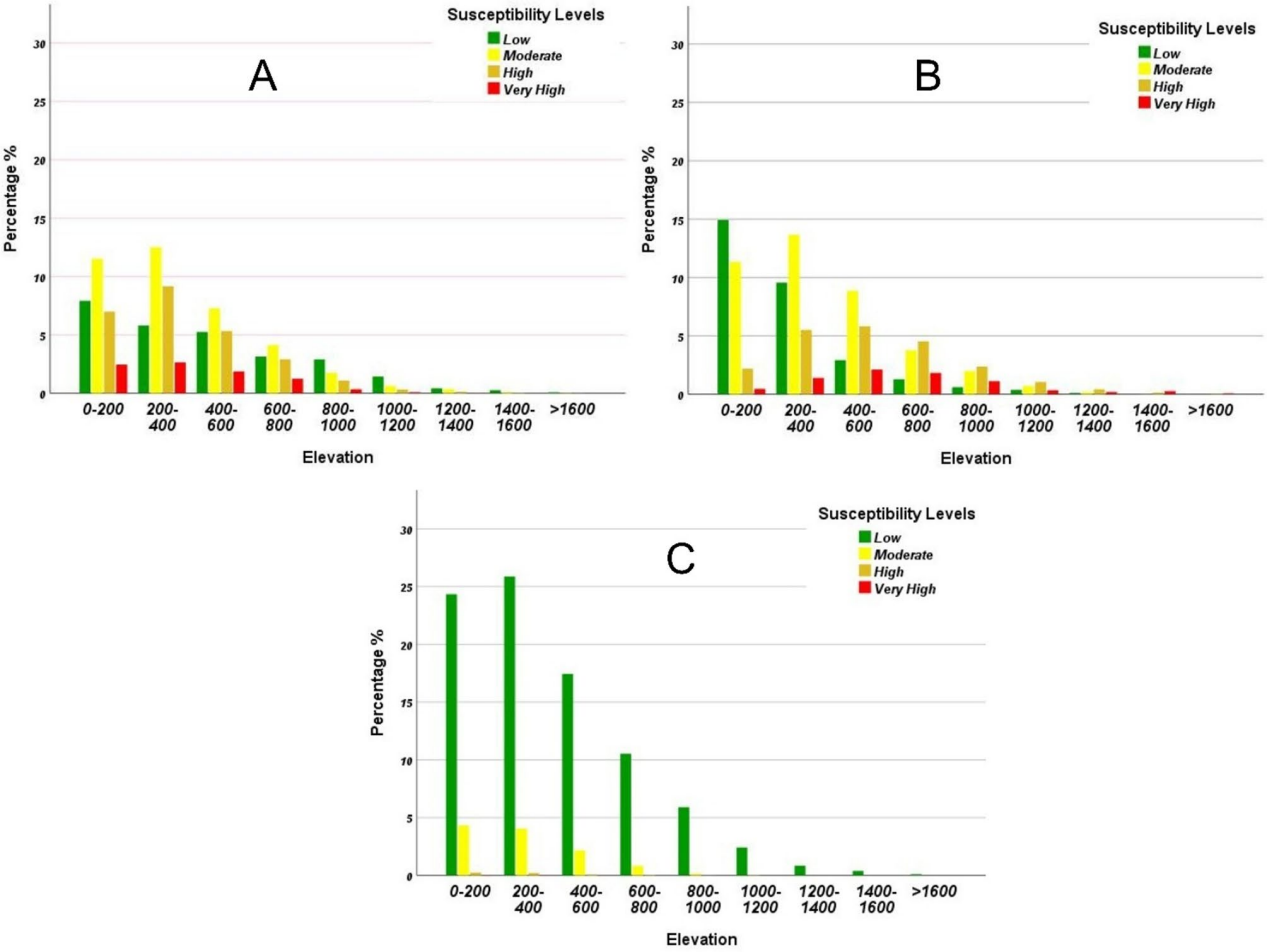
Frequency distribution of landslides susceptibility categories using different LIMs in Martil watershed study area: (**A**) Old landslides, (**B**) Inactive-young LIM, (**C**) new active LIM.

Statistical proof for this observation is presented in Fig. 12, where one can clearly see the shift in the topographic position of high susceptibility areas from a peak frequency at the 400–600 m elevation category in the case of the relict landslides LSM (Fig. 12A) to 200–400 for old landslides (Fig. 12B) and finally 0–200 m in the case of the active/new processes LSM (Fig. 12C). By viewing these LSMs as spatial distribution maps rather than spatial hazard ones, one can establish a clear link between the gradual abandonment of relict and Inactive-young processes due to base level fall episodes of the Quaternary times. As such, the relict mass movements are currently left behind because of their dependency on surface runoff erosion in the tight DEF valleys. These conclusions however require radiometric dating for definitive proof.

## Conclusion

To sum up, the results of this research point to the importance of not viewing the study area as just a randomly selected sampling pool but as distinct geomorphological units that need to be carefully selected to produce meaningful and useful statistical models both in terms of comparability and generalizability of the output. To achieve this outcome, one must consider the aims of their study. First of all, the morphotectonic units shaping the landscape must be identified. Then, a decision on the main objective must be taken: if the outcome is to be used for planning, then the study area selection must be tied to the same morphotectonic unit to generate reproduceable outcomes especially when different landslide types are involved (relict, old and new processes that significantly differ from one unit to the other). This is the case for Tetouan province and Tetouan map in this study where the output LSMs are almost identical for the overlapping areas, with very similar AUC values (10% difference at maximum) and landslides probability assessments (5 to 10 points difference at maximum). Such models are also more useful for planning stabilization and prevention works as they provide solid morpho-structural explanations to the observed statistical associations. Nevertheless, if comparing multiple landscapes is the objective, then using a larger study area with multiples morphostructural elements provides a good basis for comparative purposes, as the extent of the study area allows comparing the impact of conditioning factor variability on landslides occurrence, across diverse environments. This may however lead to a significant decrease in accuracy values as is the case for Tetouan province, with AUC for this study area being 5 to 8 points lower than the other two areas. Failure to recognize these issues significantly compromises the interpretability of the results in both of the above scenarios. All in all, and despite the promising findings presented above, further research may help shed more light on this issue as data covering three, four or even five morphotectonic units can be used to further assess the impact of study area heterogeneity on probabilistic models. In fact, we believe that solving this issue may initiate a new era in LSM studies where the aims of landslides probabilistic modelling dictate the appropriate research approach and strategy.

## Data Availability

The datasets used and/or analyzed during the current study available from the corresponding author on reasonable request.
